# Three-year-old traumatic liver injury patient treated successfully using transcatheter arterial embolization

**DOI:** 10.1016/j.ijscr.2020.04.036

**Published:** 2020-05-11

**Authors:** Atsuyoshi Iida, Tsuyoshi Ryuko, Masaichi Kemmotsu, Hiroaki Ishii, Hiromichi Naito, Atsunori Nakao

**Affiliations:** a Critical Care Medical Center, Okayama Red Cross Hospital, Okayama, Japan; b Department of Surgery, Okayama Red Cross Hospital, Okayama, Japan; c Department of Radiology, Okayama Red Cross Hospital, Okayama, Japan; d Department of Emergency, Critical Care, and Disaster Medicine, Okayama University Graduate School of Medicine, Dentistry and Pharmaceutical Sciences, Japan

**Keywords:** Hepatic injury, Non-operative management, Transarterial embolization, Pediatric, Case report

## Abstract

**Introduction:**

Liver injury is the most vulnerable to blunt abdominal trauma. Diagnostic evaluation and treatment of blunt liver trauma in children have changed essentially over the last decades.

**Presentation of case:**

A 3-year-old girl, weighing 10 kg was run over by a car and admitted to our hospital.　Due to the liver injury and increased intra-abdominal hemorrhage confirmed by computed tomography, emergent transcatheter arterial embolization (TAE) was performed. Hemostasis was successfully obtained without complications. The patient had a good postoperative course and was discharged on the 9th day after admission.

**Discussion:**

To the best of our knowledge, this case is the youngest and lowest weight emergency TAE success cases of childhood liver injury. TAE is an alternative to laparotomy and a useful procedure to accomplish nonsurgical management in adult who are hemodynamically stable and have no other associated injury requiring laparotomy. On the other hand, TAE is considered to have some complications in child cases because of the small diameter of the artery and the tendency to spasm. Our case showed that TAE can be a safe option for emergency hemostasis in pediatric trauma cases weighing 10 kg.

**Conclusion:**

Emergency physicians must be aware that radiological intervention is an important adjunct to management of childhood liver injury.

## Highlights

• Liver injury is the most vulnerable to blunt abdominal trauma.
• Treatment of blunt liver trauma in children have changed essentially over the last decades.
• TAE hemostasis was successfully administered to a 3-year-old child with liver injury.
• TAE can be a safe option for emergency hemostasis in pediatric trauma cases.

## 1 Introduction

Blunt liver injury is the most commonly encountered pediatric abdominal trauma and a leading cause of mortality and morbidity in children, since the flexible pediatric rib cage grants transmission of blunt force energy to the liver [1,2]. In most cases, liver trauma may be a consequence of a massive violent impact and thus is usually combined with other injuries. Therefore, liver injury management, particularly in pediatric polytrauma, challenges emergency physicians and pediatric surgeons. Hemorrhage resulting from hepatic injury is the major cause of mortality and morbidity. Recently, the hemostatic treatment protocol for blunt hepatic trauma has shifted significantly from an early mandatory surgical strategy to a more conservative, non-surgical approach. In most reported case series of pediatric hepatic injury, children have been conservatively treated without surgery or interventional radiology [2].

Transcatheter arterial embolization (TAE) for liver trauma bleeding has been well established as the standard treatment for blunt hepatic injury in adults, allowing control of hemorrhage and organ preservation. However, despite recent substantial progress in catheter technology and diagnostic imaging, experience with TAE to control ongoing hemorrhage associated with pediatric blunt liver injuries is still limited.

Herein, we report a case of a three-year-old girl with traumatic liver injury successfully treated with emergency TAE. To the best of our knowledge, our case is the youngest and lowest weight patient with liver injury to ever be reported. TAE for infants weighing 10 kg has rarely been reported since the smaller pediatric artery size presents technical difficulties and concerns regarding procedure-related complications. We also discuss the usefulness of emergency TAE as an initial therapeutic strategy for blunt hepatic injury and review the literature regarding indications or technical aspects. Sharing our experience may help raise awareness among emergency physicians of TAE as an alternative therapeutic strategy for blunt hepatic trauma in children.

This work has been reported in line with the SCARE criteria [3].

## 2 Case report

A previously healthy three-year-old girl weighing 10 kg was brought to our emergency department by her mother and admitted 15 min after being hit and run over by a wagon-type car. The initial examination showed her airway was patent without disturbance of consciousness. There were no signs of shock, such as wet or cold sweat on the skin. Her vital signs at the time of the visit were as follows; oxygen saturation in room air, 99 %; heart rate, 130 beats/min; blood pressure, 112/51 mmHg; Glasgow Coma Scale, 15; and body temperature, 37.7 °C. Her pupils were round with anisocoria and prompt light reflex.

Focused assessment with sonography for trauma at the time of admission demonstrated no intraabdominal fluid. Tire marks from her right shoulder to the abdomen were noted. Abdominal physical examination revealed muscle defense and tenderness on her abdomen. No limb paralysis was noted. Pelvic radiograph was unremarkable. Plain computed tomography (CT) taken 15 min after arrival showed right lung contusion, left pneumothorax, and irregular linear or branching low-attenuation areas in the liver, indicating liver laceration. Intraperitoneal fluid was not noted. Blood tests revealed a white blood cell count of 22,400/mm^3^, hemoglobin of 11.8 g/dl, hematocrit of 37.1 %, red blood cell count of 4460 × 10^3^/mm^3^, and platelet count of 434 × 10^3^/mm^3^. Blood chemistry test showed an elevation of leaking liver enzymes; 1151 IU/L aspartate aminotransferase and 599 IU/L alanine aminotransferase.

**Fig. 1:**
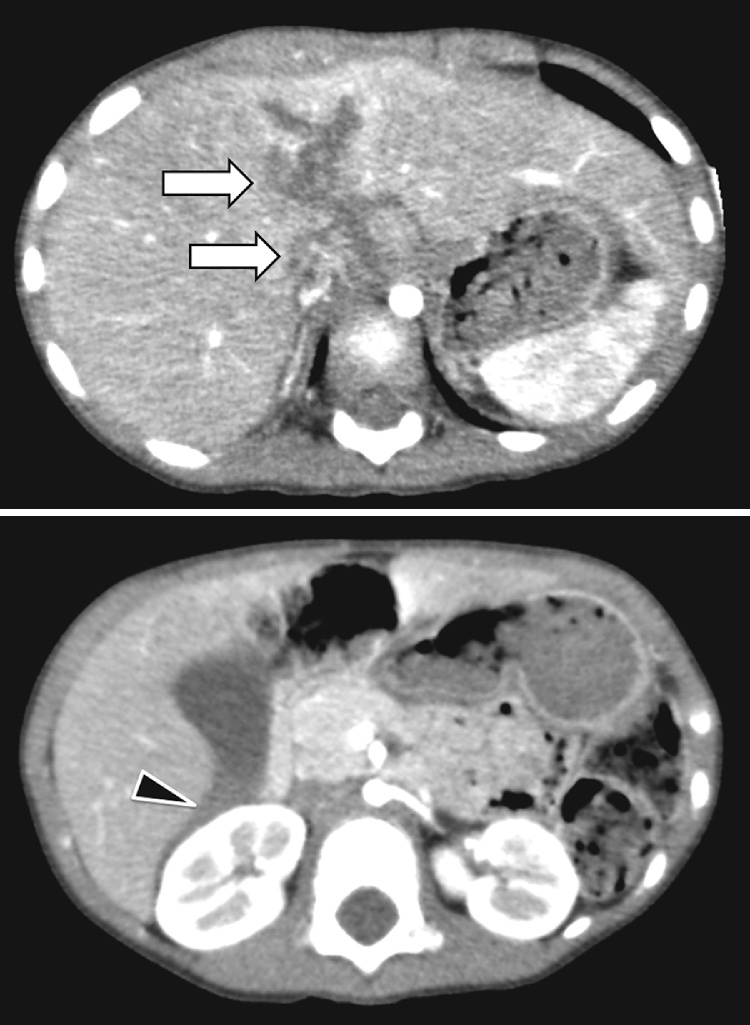
Enhanced CT one hour after admission. a) AAST Grade 4 liver injury b) Fluid collection was observed in Morison’s pouch.

**Fig. 2:**
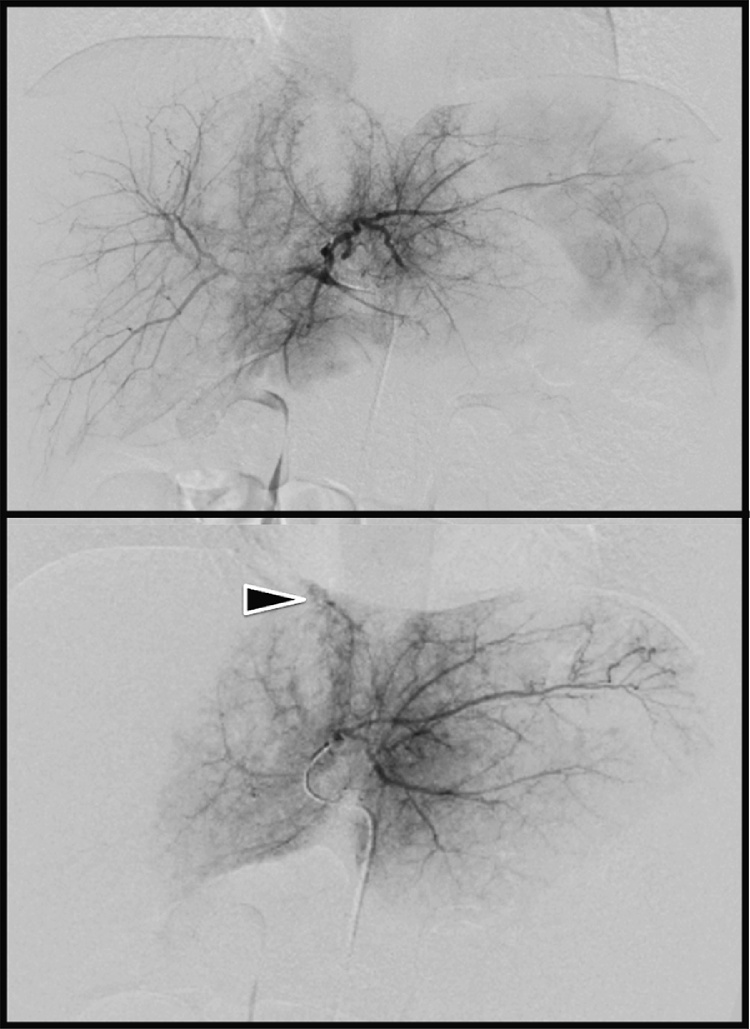
Angiography of the hepatic artery. a) Common hepatic arteriography showed no obvious bleeding points. b) Selective angiography of the left hepatic artery suggested bleeding from A4.

The patient was carefully examined with fluid injection and followed up on one hour after admission. The pneumothorax was conservatively treated. Although the patient remained hemodynamically stable, sonography detected a small amount of fluid in the Morison's pouch. A declining hemoglobin level, (9.3 g/dl) compared to that one hour prior was noticed. Contrast-enhanced CT revealed extravasation in the middle hepatic lobe associated with hemoperitoneum (Fig. 1). There was no pneumothorax or hemothorax. Based on the clinical diagnosis of active bleeding, TAE was performed for the purpose of hemostasis of active bleeding under general anesthesia with endotracheal intubation. After a 4-Fr sheath was placed in the right femoral artery, the celiac artery and common hepatic artery were visualized. When the left hepatic artery was selectively imaged using a smaller-diameter catheter, the active contrast extravasation was confirmed from a branch of the middle hepatic artery. Since selective embolization was difficult anatomically, the left hepatic artery was embolized using gelatin sponge particles (Serescue®, Nipponkayaku) (Fig. 2). Throughout TAE, her vital signs remained stable with a heart rate of 114 beats/min and blood pressure of 93/50 mmHg. Her condition stabilized overnight and the following day she was extubated. The patient was transferred to the general ward with normal liver function on the fifth day. As her clinical course was uneventful, she was discharged from the hospital on the ninth day after admission. So far (follow-up period of eight weeks after the event), the patient has been well without complications such as biloma.

## 3 Discussion

Non-operative management has generally been considered the standard therapy for clinically stable children with blunt liver injuries [2,4]. Presumably, improvement of pediatric resuscitation modalities, as well as the increasing accessibility of pediatric intensive care centers, may have supported the progressive success of non-surgical management.

Despite the current low number of reports of failure with non-surgical management, non-operative management is unsuccessful in a significant number of patients. Emergency physicians should be aware that management of hepatic injury in children should not only be based on the physiologic response because children have a strong physiological compensatory action; blood pressure decreases only after more lethal hemorrhage develops compared to adults [5]. Therefore, the decision to perform hemostasis should be made before circulatory dynamics deteriorate. Our patient showed signs of ongoing hemorrhage, such as declining hemoglobin and hematocrit levels, but hadn’t yet acquired hemodynamic compromise.

In our patient, active hemorrhage after blunt hepatic trauma was found during initial phase contrast-enhanced CT as focal high-attenuation areas showing an agglomeration of extravasated contrast secondary to arterial bleeding. Active contrast extravasation on early CT scan independently predicted the failure of non-surgical management as well as pseudoaneurysm formation, regardless of injury grade [6]. Like adult patients, early TAE in children with bleeding from hepatic artery injury may be an important adjunct to non-surgical management of hepatic traumatic injuries. The early decision to perform TAE, although the patient was hemodynamically stable, contributed to achievement of a favorable outcome [4].

However, clinical experiences with pediatric TAE have been limited and reported in only sporadic case series. The procedure is more technically difficult in young cases because of the small size of the affected arteries. Possible complications from embolization and angiography in children are similar to those in adults and include catheter or guidewire-related arterial injury, arterial puncture site hematoma, target organ ischemia, contrast nephropathy, and non-target organ embolization [7].

A review of pediatric angiography performed in trauma showed minor complication rates ranging from 0% to 4% [8]. As most reported complications were hematomas at the arterial puncture site, arterial puncture was performed using an ultrasonographic guide to avoid complication in our patient. Liver ischemia after TAE is uncommon because of redundancies in the hepatic circulation [9]. Thus, TAE should be considered a useful and safe adjunct when caring for injured children [8].

Emergency physicians must be aware that TAE is a time-consuming procedure. To ensure safety during TAE, pediatric patients should be hemodynamically stable before the procedure. Access to a pediatric anesthesiologist in the interventional radiology suite allows for continued resuscitation during the treatment. The operating room staff and surgeons should be on standby in case of sudden clinical deterioration. At some healthcare centers, sophisticated treatment protocols have been developed and interventional procedures can now be conducted in a fluoroscopy-capable hybrid operating room, which further enhances the procedure’s safety [10]. Our institute is not a pediatric specialized trauma center. A study on 2011–2012 National Trauma Data Bank cases showed that TAE was performed for 22/1449 patients (1.51 %) adult trauma centers and 12/1418 patients (0.84 %) in pediatric trauma centers, respectively [11]. Since highly specialized and dedicated radiological personnel may not be always available, TAE for pediatric patients should not be limited to pediatric trauma centers. Of note, physicians must concern the risk of radiation, since children are more sensitive to radiation and have a longer life expectancy than adults. Unnecessary radiation exposure should be avoided.

In conclusion, our experience demonstrates that TAE can be safely performed for pediatric trauma patients weighing 10 kg. Contrast-enhanced CT can help accurately identify hepatic parenchymal injuries with contrast extravasation and gauge the degree of hemoperitoneum, as well as associated injuries. Emergency physicians should consider TAE as a therapeutic option for small children with blunt liver injury, even if the patient is hemodynamically stable.

## Conflict of interest

All authors of this manuscript declare no conflicts of interests.

## Sources of funding

No funding support was given for this study.

## Ethical approval

This case study was approved by the ethical committee of Okayama University.

## Consent

Written informed consent was obtained from the patient’s guardian for publication of this case report and accompanying images. A copy of the written consent is available for review by the Editor-in-Chief of this journal on request.

## Author contribution

Atsuyoshi Iida, Tsuyoshi Ryuko, Masaichi Kemmotsu, and Hiromichi Naito contributed to the study design, data collections, data analysis, writing and review. Hiroaki Ishii performed the transarterial embolization. Atsunori Nakao contributed to the data collections and review.

All authors have read and approved the final manuscript.

## Registration of research studies

Not applicable.

## Guarantor

Hiromichi Naito (Corresponding author).

## Provenance and peer review

Not commissioned, externally peer-reviewed.
